# Burden of Surgical Treatment for the Management of Cervical Myelopathy in Mucopolysaccharidoses: A Systematic Review

**DOI:** 10.3390/brainsci13010048

**Published:** 2022-12-26

**Authors:** Roberta Costanzo, Lapo Bonosi, Massimiliano Porzio, Federica Paolini, Lara Brunasso, Andrea Evier Giovannini, Manikon Poullay Silven, Giuseppe Roberto Giammalva, Giuseppe Emmanuele Umana, Gianluca Scalia, Domenico Gerardo Iacopino, Rosario Maugeri

**Affiliations:** 1 Neurosurgical Clinic, AOUP “Paolo Giaccone”, Post Graduate Residency Program in Neurologic Surgery, Department of Biomedicine Neurosciences and Advanced Diagnostics, School of Medicine, University of Palermo, 90127 Palermo, Italy; 2 Department of Neurosurgery, Cannizzaro Hospital, Trauma Center, Gamma Knife Center, 95125 Catania, Italy; 3 Head and Neck Surgery Department, Neurosurgery Unit, Garibaldi Hospital, 95123 Catania, Italy

**Keywords:** mucopolysaccharidoses, cervical, myelopathy, instability

## Abstract

Mucopolysaccharidoses (MPSs) are a rare group of heterogeneous genetic and metabolic disorders, caused by loss of functions of several enzymes that are involved in glycosaminoglycan catabolism. Their progressive accumulations in cells, tissues, and consequently, organs lead to several clinical manifestations, such as musculoskeletal involvement. Indeed, the most common manifestation in the central nervous system is represented by cervical spinal stenosis due to bony alterations or dural thickening. Cervical involvement can commonly cause myelopathy and instability exerting severe symptoms. A prompt diagnosis and treatment of the aforementioned conditions is mandatory to ensure a better quality of life in patients with such debilitating disorders. Nevertheless, a clear consensus about their management (i.e., surgical or not) is still lacking, leading to an inevitable delay. This review aims to investigate and discuss the main causes of myelopathy in patients with mucopolysaccharidoses, available therapeutic strategies, and the impact and role of surgery on the neurological outcome.

## 1. Introduction

Mucopolysaccharidoses (MPSs) are a rare group of heterogeneous genetic disorders involving glycosaminoglycan (GAGs)—or mucopolysaccharides—catabolism, caused by mutations inactivating lysosomal enzymes that are required for GAGs degradation [1].

The incidence rate of MPSs is around 1:22,000–25,000 live births. The overall incidence of specific MPSs disorders varies between ethnic groups [2,3].

GAGs are a heterogeneous family of linear, complex, and highly sulfated polysaccharides which constitute many proteins and proteoglycans, which are functional units of human connective tissue [2,4]. GAGs degradation is performed by specific enzymes, exoglycosidases, and sulfatases, acting in succession; therefore, a lack of their function leads to altered GAGs degradation and overloading in urine, blood, and cerebrospinal fluid (CSF) [1,3,5]. MPSs pathophysiological mechanisms hence regard a block of the lysosomal degradative function, leading to GAGs accumulation and progressive cellular damage. Multiple tissues and organs can be involved [1]. Nowadays, 7 types and 13 subgroups of MPSs syndromes have been identified, which are caused by deficiencies in one or multiple of eleven different enzymes [3].

The symptoms and their severity widely diverge between the wide-ranging set of these syndromes. Musculoskeletal involvement is a common manifestation. In particular, the thoracolumbar spine and the cranio-cervical junction with consequent cervical myelopathy represent the two main locations of spinal cord compression [2]. Early diagnosis and treatment are the only chance to ensure stable clinical status and a better quality of life. In this regard, neurosurgeons play a crucial role in patients’ therapeutic strategy. Hence, in this systematic review the authors searched the literature to investigate and discuss the main causes of myelopathy in patients with mucopolysaccharidoses, available therapeutic strategies, and the impact of surgery on the neurological outcome, considering new enzyme replacement therapies.

## 2. Materials and Methods

**Figure 1:**
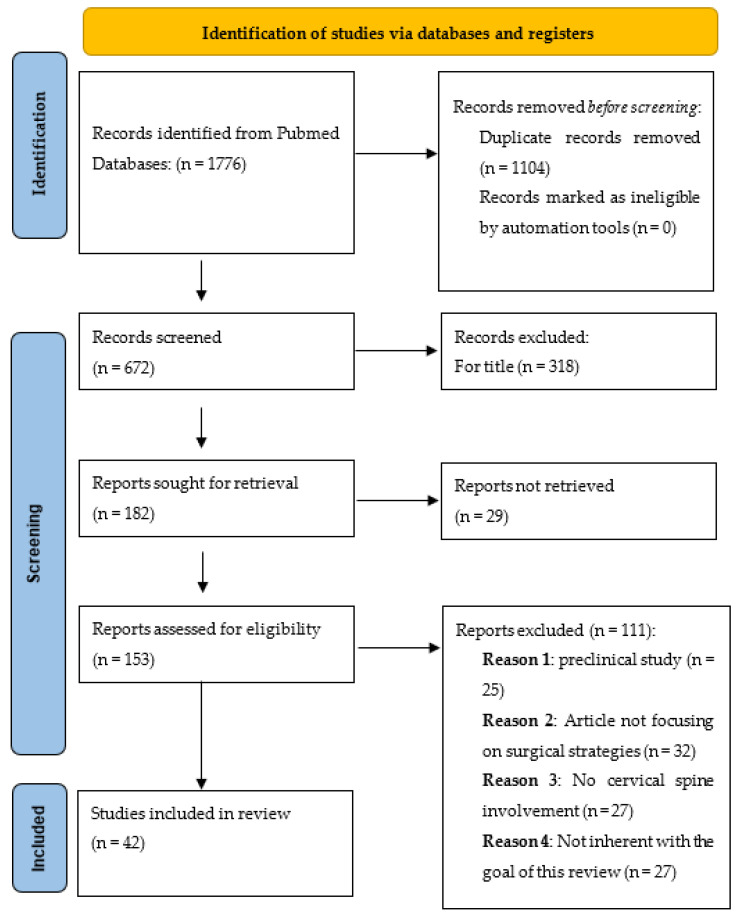
PRISMA flow diagram.

A systematic literature review was conducted following the preferred reporting Items for systematic reviews and meta-analyses guidelines (PRISMA) (Figure 1). The authors performed a broad systematic literature search in the PubMed database for all studies investigating the cervical spine involvement in patients with mucopolysaccharidosis (MPS), both clinically and surgically. The authors made systematic searches for studies that were published up to June 2022, without backward limits, using the following Mesh and free text terms as follows: “Mucopolysaccharidosis”, “Cervical Spine”, “Spinal Cord”, “Cervical”, “Myelopathy”, “Spinal Cord Compression”, “Cervical Stenosis”, Cervical disease”, “Hurler”, “Scheie”, “Maroteaux”, “Morquio”, “Hunter”, “Sly”, and “Sanfilippo” combined using Boolean operators “AND” and “OR”. To avoid the potential omission of relevant studies, the authors also manually screened reference lists of articles that were included and previous systematic reviews and meta-analyses regarding cervical spine involvement in MPS diseases. Duplicate articles were excluded using Microsoft Excel 16.37 (Redmond, WA, USA).

### 2.1. Study Selection

The research strategy initially relied on title and abstract analysis. The article’s full text was retrieved for further investigation if the title and abstract met the inclusion criteria. Two authors (M.P. and L.B.) independently assessed eligibility based on “a priori” criteria. The data collection process was conducted without using any automated tools. No ethical approval was required for this study.

#### 2.1.1. Eligibility Criteria

The articles were selected according to the following inclusion criteria:

- Full article in English
- Clinical studies (case reports, case series, retrospective/prospective studies)
- Patients that were affected by MPS without regarding the type of disease
- Studies focusing on patients with cervical spine involvement and consequent cervical myelopathy

The exclusion Criteria were:

- Articles that were not in English
- Article that did not focus on surgical treatment
- Patients that were not affected by MPS
- Patients with spine involvement other than cervical segment
- Patients with cervical spine involvement without clinical signs of cervical myelopathy

#### 2.1.2. Data Extraction

According to the aforementioned criteria, all of the articles were identified independently by two reviewers (M.P and L.B). The extracted data included the following: author, publication time, country, study design, patient characteristics (number of patients, age, and gender), MPS typology, cervical spine level, etiology of myelopathy and clinical manifestations, surgical treatment modalities, clinical outcomes, additional treatment, adverse events (AEs), other localizations of MPS disease, and follow-up time.

## 3. Results

### 3.1. Data Selection

A total of 1176 articles were identified on the PubMed database. After duplicates removal (1104), based on a screening by title and abstract, we excluded further 519 articles.

There were 25 articles that were not included because they were based on preclinical studies, 32 were excluded because they were not focused on surgical strategies, 27 because they did not mention a cervical spine involvement, and finally, 27 due to incompatibility with our eligibility criteria. A total of 42 articles were included in the present systematic review. The patients’ demographics are shown in Table 1.

#### Patients’ Demographic and Clinical Evidence

**Figure 2:**
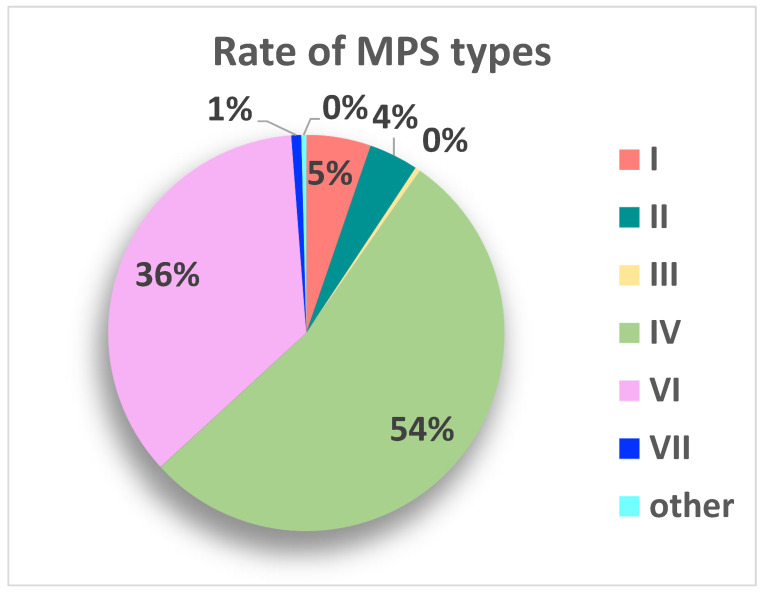
Rate of MPS type that is associated to cervical myelopathy (Other: one patient has an intermediate phenotype V/VI).

A total of 247 patients were collected, 43.13% females (n =66) and 56.86% males (n = 87). The mean age of the patients at the time of myelopathy diagnosis was about 11.01 ± 8.80 years. The main types of mucopolysaccharidosis that were involved were: 13 patients with Type I (5.26%), 10 patients with Type II (4.04%), 1 patient with Type III (0.40%), 132 patients with Type IV (53.44%), 88 patients with Type VI (35.62%), 2 patients with Type VII (0.80%), 1 patient with the intermediate phenotype V-VI (0.40%); the rates are shown in Figure 2.

**Figure 3:**
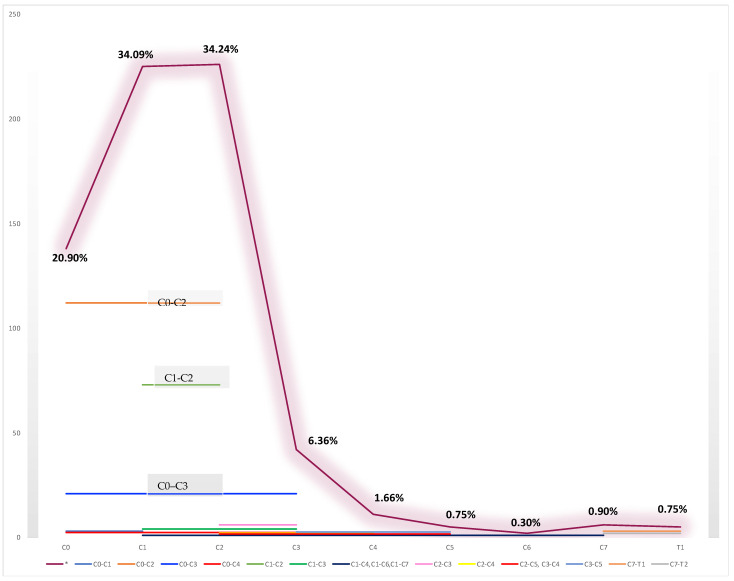
The purple line (*) shows the rate of patients that were affected by spinal cord compression related to the cervical levels, with a clear prevalence of the cranio-vertebral junction (C0–C2).

For each type of Mucopolysaccharidosis, the main level of cervical compression is shown in Table 2. The main cervical spine tracts that were involved were: 3 patients C0–C1 (1.21%); 112 patients C0–C2 (45.34%); 21 patients C0–C3 (8.50%); 2 patients C0–C4 (0.80%); 7 patients C1 (2.83%); 73 patients C1–C2 (29.55%); 4 patients C1–C3 (1.61%); 1 patient C1–C4 (0.40%); 1 patient C1–C6 (0.40%); 1 patient C1–C7 (0.40%); 2 patients C2 (0.80%); 6 patients C2-C3 (2.42%); 2 patients C2–C4 (0.80%); 1 patient C2–C5 (0.40%); 1 patient C3–C4 (0.40%); 2 patients C3–C5 (0.80%); 3 patients C7–T1 (1.21%); 2 patients C7–T2 (0.80%); and 3 N/A (1.21%) (Figure 3).

In Figure 3, we show the main cervical spine level that is involved with a clear prevalence of the cranio-vertebral junction (C0–C2), including a total of 197 patients (79.73%).

The different etiologies of MPS cervical compression were investigated and 15.38% (n = 38) of the cases were related to flavum ligamentum hypertrophy and dura mater thickening, 14.57% (n = 36) cases were due to atlanto-axial instability, and 65.58% (n = 162) of cases were due to the association of both conditions. Moreover, 39.67% of patients that were included in this review that were affected by MPS Type IV cervical myelopathy were related to atlanto-axial instability, while in 23.88% of patients with MPS Type VI were due to cervical stenosis. Patients that were included in this review presented clinical symptoms of motor disturbances, ranging from 78.07% of myelopathy symptoms, to 70.17% of paresis, 1.31% of plegia, and 47.80% alteration or/and inability to walk. Surgical treatment of choice was osteo-ligamentous decompression in 92.30% of patients (n = 228), and was associated to fusion in 45.17% of cases (n = 103). The median follow-up was about 61.31 ± 65.07 months, with a comprehensive evaluation of patients’ postoperative neurological status. The clinical outcome improved in 90.04% (n = 199), deteriorated in 4.07% (n = 9), while was stable in 5.42% of cases (n = 12). Furthermore, according to our literature review, among 10 studies (27.02%) surgery was delayed, with a mean time of 2.9 years after diagnosis of cervical myelopathy. In the other 27 studies (72.97%), surgery was planned immediately after the diagnosis of cervical myelopathy.

## 4. Discussion

Mucopolysaccharidoses are a heterogeneous and wide-ranging set of genetic syndromes that are characterized by progressive and debilitating diffuse manifestations, with a high rate of morbidity and mortality for affected patients. Even so, some key manifestations, such as musculo-skeletal spinal features, are commonly shared among some types of MPSs.

Once a clinical diagnosis is made, typically within the first years of life, based on the phenotype of these patients, neurologic assessment, and neuroimaging features, a following biochemical diagnosis is conducted. Moreover, genetic examinations of the patients and their relatives can be performed before birth as well [25,47].

The cervical spine is often affected by radiological abnormalities, resulting in spinal cord compression, secondary to malformations of the spine and the CVJ, and/or deposit of GAGs in the soft tissues adjacent the spinal cord. The natural history of spinal cord compression sequentially encompasses local ischemia, edema, neuronal cell death, with subsequent myelomalacia.

Notably, CVJ instability is a hallmark feature in many different diseases and is mostly recognized in MPS Type I, IV, and VI, while cervical canal stenosis can be often found in the majority of MPSs, mainly in Type I, II, VI, and VII [45,48].

In addition, patients with classical MPS Type IV A may present severe skeletal dysplasia and growth disturbances that affect the respiratory function [44].

Clinical findings are often represented by cervical myelopathy symptoms, arising from long tracts compression, including bilateral motor deficits, painful paresthesia, sphincter disturbances, and hyperreflexia. Thus, in this systematic review, all of the studies that were selected and evaluated involved patients with cervical myelopathy, enlightening how this condition is a common and recurring characteristic of these genetic disorders. Indeed, from a neurosurgical point of view, plain and dynamic radiographs, CT, and MR scans play an important role in MPSs management.

Typical radiological musculoskeletal features that are observed in MPSs cervical spine are:

- Odontoid hypoplasia and peri-odontoid soft tissue masses arising from GAGs deposits posterior to the odontoid process. Atlantoaxial subluxation and occipito-atlantoaxial instability, sustained by odontoid hypoplasia and laxity of the transverse and alar ligaments is less commonly observed in MPSs [47].
- Reactive hypertrophy of the ligamentum flavum
- Dural thickening
- Protrusion and/or intervertebral disc herniations [2,44].
- Basilar invagination or enlarged morphology of the mastoid processes [44].

All these abnormalities can lead to severe spinal canal stenosis and related alterations, with or without abnormally high signal intensity zone in T2-weighted MRI sequences, otherwise the typical radiological signs that are associated with cervical myelopathy [49,50].

It has been observed that patients with MPS Type VI tend to develop posterior longitudinal ligament hypertrophy, with a consequent cervical spinal stenosis. On the other hand, MPS Type IV is characterized by early development of cervical instability, due to the accumulation of keratan sulfate (KS) and chondroitin-6-sulfate, that alter cartilage and bone development, as reported in Table 1 [25,44].

According to Horovitz et al., MRI studies have documented that a sagittal diameter of 13 mm may be associated with neurological symptoms or signs. Indeed, there may be dynamic spinal canal stenosis during flexion-extension excursions—without compression of the spinal cord in a neutral cervical position—since the antero-posterior diameter of the spinal cord is a few millimeters narrower than the canal itself [25].

With regards to the radiological study of MPSs, a plain radiograph in two projections is generally first accomplished to evaluate spinal involvement, followed by flexion-extension lateral radiographs. CT scans allow a more accurate characterization of bony changes and abnormality; it is nowadays considered as the gold standard to achieve a better characterization of the odontoid process and atlanto-axial articulation. Furthermore, MRI can be used to adequately assess the spinal cord’s involvement signs. Routine MRI assessment instead, and even flexion-extension dynamic MRI, can be used in the monitoring of spinal cord compression, spinal canal stenosis, and cervical myelopathy [2,34].

Somatosensory evoked potentials (SSEP) and motor evoked potentials (MEP) analysis seems helpful in patients’ follow-up to monitor the peri-operative neurological status in MPSs with signs of spinal cord compression [25].

Among all the available tools to detect spinal cord alterations, transcranial magnetic stimulation has proven to be useful in the evaluation of spinal cord disease, especially at the first stages of neural compression, as it may be deemed as a functional counterpart of neuroimaging, and an extension of clinical assessment [51,52].

To gain a better understanding of the main features of MPSs, here we focus on a study by Montaño et al., including 143 patients with MPS VII, that were diagnosed in 30 countries. Patients with more severe phenotypes had short stature and increased features of skeletal dysplasia. A total of 90% of patients with MPS VII presented with skeletal dysplasia such as vertebral breaking and hypoplastic odontoid process, with atlantoaxial instability. These patients have an increased risk of cervical dislocation, even from minor trauma or intubation maneuvers [53].

Another interesting case that was reported by Utsunomiya et al., has shown a child with MPS Type IVA that during the second year of enzyme replacement therapy (ERT) developed a compression of the spinal cord at the C1 level with a consequent instability of the atlantoaxial joint. Although fusion of the occipito-cervical spine with C1 posterior decompression is generally performed for patients with MPS Type IV A, decompression surgery was performed at first, and only later, fusion with instrumentation was performed because the bone structures were initially too small due to the young age of the patient [44]. This case has opened the demanding role of timing in the surgical treatment of these disorders, especially during ERT therapy. Indeed, after the first year of treatment, an improvement of posture has been evaluated leading to a late development of spinal stenosis that is probably related to a difficult capacity to reach bone tissues by the ERT agent.

The main role of surgery in these patients is to determine a neurological improvement, whenever feasible, and/or prevent further neurological impairment, ultimately allowing for a better quality of life. However, given the heterogeneity of the syndromic manifestations that are linked to different types of MPSs, a clear consensus regarding surgical indications is lacking.

As previously explained, one of the main causes of cervical myelopathy can be related to a narrow osseous neural canal due to dural thickening [9,54].

Indeed, the study by Ballenger et al. was the first study of Hunter syndrome complicated by cervical myelopathy due to thickened meninges which is also implicated in intracranial subarachnoid blockade of CSF, thus promoting communicating hydrocephalus. In this case, early cervical decompression was found to be promising for stabilization and improvement of myelopathic symptoms [9].

Nevertheless, preventive treatment in asymptomatic patients with neither clinical or radiologic evidence of myelopathy is still a matter of debate.

On considering the natural course of MPSs, prophylactic fusion of the cervical spine has been recommended to prevent cervical myelopathy. Early surgery is proven to obtain better neurological outcomes, preventing otherwise progressive neurological impairment [29]. As shown in Table 1, 90.04% of the patients reported a significant clinical improvement after surgery.

Upper cervical fusion is recommended when radiographic signs of progressive spinal instability and/or compression are present, even without neurologic impairment, to prevent spinal cord compression and improve neurologic outcome [29].

Dede et al. observed that plain radiographs in children that were affected by Morquio syndrome underestimated the real diameter of the spinal canal. In fact, due to the presence of an unossified odontoid tip and soft tissue GAGs deposition posterior to the odontoid, an MRI study of the cervical spine is essential in children with Morquio syndrome with neural changes and instability, highly suggestive of subsequent spinal cord compression. [29].

According to some authors, surgical preventive treatment should be recommended in all MPS IV (Morquio syndrome) patients with MRI evidence of CVJ stenosis and instability, with canal narrowing of >50% [38]. Moreover, clinical evidence of acute or progressive myelopathy due to spinal cord compression at the CVJ represents a clear surgical indication. First, surgical decompression could, therefore, lead to a neurological function improvement even in long-term follow-up [29,38].

Otherwise, patients with severe compression at CVJ may benefit from anterior transoral decompression and posterior fusion, as it could possibly give a better and prompter neurological recovery [11,55].

Laminoplasty can also be considered a safe and effective surgical procedure allowing for a better preservation of the cervical range of motion, thus preventing kyphotic transformation after surgery. Nevertheless, it cannot be performed in most pediatric patients due to the small dimension of the laminae, and when odontoid hypoplasia is highlighted [45].

Early decompression surgery for CVJ disease is often required in most young patients with MPS IV A Type (Morquio syndrome), when considering the aggressive natural history of the disease and high risk of acute neurologic deterioration [39].

In the literature, an interesting case of a child with MPS type IV A, affected by C3–C5 kyphotic deformity myelopathy has been documented. At first, the patient underwent conservative treatment with halo ring placement. Then, C3–C5 corpectomy was performed using a cranial bone graft between the bodies of C2 and C6, to gain anterior decompression. Finally, a halo-vest was applied.

Even the use of occipital bone grafts is reported in patients thata re affected by atlantoaxial instability to obtain further stabilization of the midcervical region [14].

## 5. Conclusions

The results of our systematic review confirm prior investigations, assessing that surgical decompression, performed with or without fusion, can be considered the most efficacious and safe surgical treatment. Above all, surgery should be considered in MPSs with neural compression from either atlanto-axial instability and soft tissue thickening in patients with clinical evidence of myelopathy and/or spinal cord compression.

The main limitation of this study concerns a selection bias since several studies regarding surgical strategies for MPSs have been excluded due to the lack of clinical data. Therefore, uniform data for pre- and post-operative clinical evaluation could not be fully extracted from all the selected studies.

Nevertheless, MPSs are rare genetic disorders that, due to the lack of clear guidelines and uniform data, require future studies and longitudinal pre- and post-operative exams for early diagnosis, accurate prognosis, and adequate surgical treatment.

## Figures and Tables

**Table 1:** Demographic and clinical data from our studies’ selection.

1	Author	Country	Study Design	N of Patients	M	F	Mean Age	MPS Type	Level of Spine Pathology	Aethiology of Myelopathy	Surgical Treatment	AEs	Additional Treatment	Clinical Outcomes	Follow Up Time after Surgery (Months)
1973	P Kennedy [6]	England	Case Report	1	0	1	16	V/VI	C1–C2	Ligamentum flavum hypertrophy & dura mater thickening	C1–C5 laminectomy	CSF leak; pseudo-myelomeningocele; then AVD	-	neurological improvement, regain walking	(seems long-term)
1974	W.G. Paulson [7]	USA	Case Report	1	0	1	55	I S	C1–C6	Ligamentum flavum hypertrophy & dura mater thickening & severe stenosis	C1–C6 laminectomy	Transient CSF leakage	-	neurological improvement	/
1975	D I Peterson [8]	California USA	Case Report	1	0	1	23	VI	C1–C3	Ligamentum flavum hypertrophy & dura mater thickening	C1–C5 laminectomy	-	-	neurological improvement, regain walking, and regain continence	(seems long-term)
1980	C E Ballenger [9]	Georgia USA	Case Report	1	1	0	24	II B	C2	ligamentum flavum hypertrophy & dura mater thickening	C1–C7 laminectomy	-	-	neurological improvement, re-gain walking and strength	N/A
1987	M Sanna [10]	Saudi Arabia/Canada	Case Report	1	0	1	16	VI	C1–C3	Ligamentum flavum hypertrophy & dura mater thickening	C1–C3 laminectomy	-	-	neurological improvement	/
1991	J Ashraf [11]	England	Case Report	1	1	0	3.5	IV	C1–C2	Atlanto-axial instability, dura mater thickening	transoral odontoidectomy; posterior decompression and fixation C0–C2	-	Preop halo traction	neurological improvement, re-gain walking and strength	/
1991	J M Stevens [12]	England	Case Series	8	5	3	9.7	IV	C1–C2	Odontoid dysplasia, atlanto-axial instability	CCJ decompression	-	2 pt: redo	limited neurological improvement	up to 120 m
1996	H Northover [13]	Netherlands	Clinical Review	22	N/A	N/A	1.5	IV	C1–C2	Odontoid dysplasia, ligamentum flavum hypertrophy & dura mater thickening	CCJ decompression and fusion	-	2 pt: fixation redo	-	(seems long-term)
1996	C B Piccirilli [14]	Michigan USA	Case Report	1	1	0	4.5	IV	C3–C5	Cervical kyphosis with stenosis	anterior corpectomy C3–C5, PLL removal, fixation with autologue graft	-	Preop halo traction	neurological improvement	-
1997	D P O’Brien [15]	England	Case Report	3	3	0	16.33	II	C0–C2	Odontoid dysplasia, ligamentum flavum hypertrophy & dura mater thickening	foramen magnum decompression, C1–C6 laminectomy	1 pt: wound infection 1 pt: CSF leakage	-	neurological improvement	-
2000	E Kachur [16]	Canada	Case Report	1	0	1	8	I H	C1–C4	Ligamentum flavum hypertrophy & dura mater thickening	C1–C5 laminectomy, foramen magnum decompression	-	-	neurological improvement	24 m
2001	J A Thorne [17]	England	Case Series	4	2	2	12	VI	1 pt: C1–C3 1 pt: C0–C3 1 pt: C1–C3 1 pt: C0–C4	Ligamentum flavum hypertrophy & dura mater thickening	Upper cercival decompression and fusion	1 pt: CSF and wound infection	3 pt: Preop halo traction	neurological improvement	36 m
2003	S A Khan [18]	England	Case Report	1	0	1	59	I H	C3–C5	Ligamentum flavum hypertrophy & dura mater thickening	C2–C4 laminectomy and fixation	-	-	neurological improvement, pain reduction	96 m
2004	R D Dickerman [19]	New York USA	Case Report	1	1	0	1.41	VII	C0–C2	Atlanto-axial instability, ligamentum flavum hypertrophy & dura mater thickening	C1–C3 laminectomy, foramen magnum decompression and fusion with autologue graft	-	Post-op halo vest	neurological improvement	-
2005	M Mut [20]	Turkey	Case Report	1	1	0	2	VI	C0–C4	Ligamentum flavum hypertrophy & dura mater thickening	Foramen magnum decompression and C1 laminectomy		After years, restenosis: C2–C4 laminectomy	neurological improvement	-
2009	S Illsinger [21]	Germany	Case Report	1	0	1	26	I S	C2	Ligamentum flavum hypertrophy & dura mater thickening	C2 laminectomy	-	ERT therapy	neurological improvement	-
2009	K K White [22]	California USA	Case Report	1	1	0	10	IV	C1–C2	Odontoid dysplasia, atlanto-axial instability	C1 laminectomy and fusion with autologue graft	-	Post-op halo vest	neurological improvement	312 m
2009	C Giussani [23]	Italy	Case Report	1	0	1	6	IV	C0–C2	Odontoid dysplasia, atlantoaxial instability	C1–C2 decompression, C0–C2 fixation	-	-	neurological improvement	36 m
2011	J K Houten [24]	N.Y. USA	Case Report	1	0	1	17	IV	C1–C2	Atlanto-axial instability, dura mater thickening	C1–C4 laminectomy, foramen magnum decompression and fusion	-	-	neurological improvement	10 m
2011	D D G Horovitz [25]	Brazil	Case Report	3	1	2	5.26	VI	C0–C2	Atlanto-axial instability, OPLL	Cervical decompression and fixation	-	3 pt: ERT	2 pt: neurological improvement 1 pt: neurological improvement and continence improvement	24 m
2011	M C Tchan [26]	Australia	Case Report	3	3	0	43.33	II	1 pt: C2–C3 1 pt: C2–C4 1 pt: C2–C4	Ligamentum flavum hypertrophy & dura mater thickening	Cervical decompression	-	idursulfase therapy	1,2: neurological improvement; 3: clinically stable	64 m
2011	D Shukla [27]	India	Case Report	1	0	1	20	IV	C0–C2	Ligamentum flavum hypertrophy & dura mater thickening	Foramen magnum decompression, C1–C2 decompression	-	-	neurological improvement	10 m
2012	CKV Tong [28]	Canada	Case Report	1	0	1	16	IV	C0–C3	Odontoid dysplasia, atlantoaxial instability, severe stenosis	C1 decompression	-	-	neurological deterioration	12 m
2013	O Dede [29]	Delaware USA	Case serie	20	N/A	N/A	5.25	IV	5 pt: C1–C2 1 pt: C0–C3 14 pt: C0–C2	Atlanto-axial instability	CCJ decompression + 5 pt: C1–C2 fusion; 1 pt: occipito-C3 fusion; 14 pt: occipito-C2 fusion		Postop halo vest. 1 pt fixation exstention to c1; 3 pt fixation exstention to c4; 2 fixation exstention to c5	neurological improvement	106 m
2013	C Möllmann [30]	Germany	Retrospective	12	N/A	N/A	7.9	IV	C0–C2	Atlanto-axial instability, ligamentum flavum hypertrophy & dura mater thickening	3 pt: foramen magnum and C1–C3 decompression and fixation; 9 pt: foramen magnum and C1–C3 decompression	-	-	neurological improvement	/
2013	C Lampe [31]	Germany	Retrospective	14	N/A	N/A	15	VI	C0–C2	Atlanto-axial instability, ligamentum flavum hypertrophy & dura mater thickening	Foramen magnum and C1–C5 decompression	-	-	neurological improvement in 46%; no neurological changes in 54%	10 m
2014	W. A.R. Baratela [32]	Delaware USA	Case report	3	1	2	6.83	IV	1 pt: C7–T1, 1 pt: C7–T1, 1 pt: C7–T2	Cervical kyphosis with stenosis	1 pt: C7–T1 laminectomy and C5–T2 fusion; 1 pt: T1–T2 laminectomy, C7–T4 fixation; 1 pt: C7–T2 laminectomy, C6–T2 fusion.		Postop halo vest; 1 pt: after 12 years, decompression and fusion T2–T7; 1 pt: redo with C2–C4 laminectomy and posterior C2–C5 fusion	neurological improvement	144 m
2015	P Vanek [33]	Czech Republic	Case report	4	3	1	12	IV	C0–C2	Atlanto-axial instability, ligamentum flavum hypertrophy & dura mater thickening	C0–C2 decompression	1 pt: deiscence	-	neurological improvement	36 m
2016	G A Solanki [34]	California USA	Retrospective	58	27	31	13.05	VI	C0–C2	Odontoid dysplasia, ligamentum flavum hypertrophy & dura mater thickening; 7 pt also atlo-axial instability	51 pt: CCJ decompression; 7 pt: CCJ decompression and fusion	1 pt: meningitis; 2 pt: deaths anesthaesia related	8 pt: redo due to restenosis	neurological improvement	55.6 m
2017	J B Eisengart [35]	Minnesota USA	Case report	1	0	1	8	I H	C1–C7	Ligamentum flavum hypertrophy & dura mater thickening	C1 laminectomy and C2–C7 laminoplasty	-	after 4 y: (progression with C1–C2 stenosis) CCJ decompression	1 pt: neurological improvement, re-gain walking and coordination 1 pt: neurological improvement, pain reduction	72 m
2017	F Vazifehdan [36]	Greece	Case report	1	1	0	33	I S	C2–C5	Ligamentum flavum hypertrophy & dura mater thickening	Laminectomy C2–C6 and fixation C1–C2–C7	-	After 6 months and return to sports and failure C7 screws: redo with C1–C2–C5–T1–T2 fixation	neurological improvement	24 m
2018	A. Broomfield [37]	Germany	Retrospective	26	N/A	N/A	6.48	IV	18 pt: C1–C2; 3 pt: N/A; 1 pt: C1; 5 pt: C2–C3	Atlanto-axial instability, ligamentum flavum hypertrophy & dura mater thickening	19 pt: fusion; 7 pt: decompression and bone graft	2 pt: bone graft failure; 1 pt:CSF leakage	8 pt: ERT; each patient had halo vest post-op	5 pt: worsening; the others: neurological improvement	84 m
2018	M Crostelli [2]	Italy	Review	1	0	1	4.6	I H	C0–C2	Ligamentum flavum hypertrophy & dura mater thickening	Occipital-C2 decompression and fixation	-	-	neurological improvement	132 m
2018	C Giussani [38]	Italy	Review	3	2	1	4.66	IVA, VI, IV	C0–C1	Ligamentum flavum hypertrophy & dura mater thickening	Occipito-C2 decompression and fusion	-	1 pt: Preop halo vest	1 pt: neurological improvement 1 pt: neurological improvement and bladder continence improvement	24 m
2018	N Williams [39]	England	Review	18	16	2	5.8	IV	C0–C3	Odontoid dysplasia, atlanto-axial instability, ligamentum flavum hypertrophy & dura mater thickening	CCJ decompression and fusion	-	6 ERT	7 neurological improvementi; 4 complete neurological restoration; 3 clinically stable; 3 worsened; 1 not identified	102 m
2018	H Krenzlin [40]	Germany	Review	15	7	8	10.8	5 IH, 5 IVA, 5 VI	C1–C2	Ligamentum flavum hypertrophy & dura mater thickening	C1–C2 laminectomy	2 tracheostomies	9 pt: reoperation due to restenosis	neurological improvement	/
2020	R Okumura [41]	Japan	Case Report	1	1	0	54	IV	C1–C2	C1 hypoplasy, atlanto-axial instability	CCJ decompression, occipito-C2–C4–C5–T1–T2–T3 fixation	-	-	neurological improvement	24 m
2020	P Kiessling [42]	Minnesota USA	Case Report	1	1	0	17	IV	C7–T2	Cervical kyphosis with stenosis	PCF decompression, C6–T2 laminectomy, occipito-T7 fixation	-	Preop and postop halo vest	neurological improvement	17 m
2020	E Moon [43]	Germany	Case Report	1	1	0	3	IV	C1–C2	Atlanto-axial instability, dura mater thickening	C1–C2 laminectomy and fixation	-	-	neurological improvement, re-gain walking and strength	8 m
2020	A Nakamura-Utsunomiya [44]	Japan	Case Report	1	1	0	3	IV	C1	Atlanto-axial instability, ligamentum flavum hypertrophy & dura mater thickening	CCJ decompression	-	ERT	neurological improvement, re-gain walking and strength	12 m
2021	H Terai [45]	Japan	Case Report	6	5	1	27.5	1 pt: I 3 pt: II 1 pt: III 1 pt:VI	1 pt: C3–C4; 5 pt: C1	OPLL	C1 laminectomy and cervical laminoplasty	-	4 ERT	neurological improvement, re-gain walking and strength	61 m
2021	Z Demartini Jr. [46]	Brazil	Case Report	1	1	0	27	IV	C7–T1	Odontoid dysplasia, atlanto-axial instability, kyphosis with severe stenosis	CCJ decompression and fixation	died for tracheal stenosis	-	neurological improvement, re-gain walking and strength	-

M-male, F-female. MPS type: Mucopolysaccharidosis type, AEs: adverse events; CSF leak: cerebrospinal fluid leak; AVD should become VAS: ventriculoatrial shunt; N/A not available; CCJ cranio-cervical junction; PLL posterior longitudinal ligament; ERT: enzyme replacement therapy; PCF posterior cervical decompression and fusion.

**Table 2:** Main cervical level that was involved for each type of mucopolysaccharidosis.

	N Tot	Main Level Involved	%	n
I	13	C2	84.61	11
II	10	C2	70.00	7
III	1	C3–C4	100.00	119
IV	132	C2	90.15	119
VI	88	C1	100.00	88
VII	2	C1	100.00	2

## Data Availability

Not applicable.
